# Fine mapping and identification of causal alleles at the *Ur-11* locus controlling rust resistance in common bean (*Phaseolus vulgaris* L.)

**DOI:** 10.1007/s00122-025-04836-9

**Published:** 2025-02-24

**Authors:** Mohammad Erfatpour, Kristin J. Simons, Jayanta Roy, Jose C. Figueroa-Cerna, Rian Lee, James Beaver, Phillip E. McClean, Juan M. Osorno

**Affiliations:** 1 Department of Plant Sciences, North Dakota State University, Fargo, ND 58108 USA; 2 Department of Plant Agriculture, University of Guelph, Guelph, ON N1G 2W1 Canada; 3 Carrington Research Extension Center, North Dakota State University, Carrington, ND 58421 USA; 4 Department of Agroenvironmental Sciences, University of Puerto Rico, Mayaguez, PR 00680 USA

## Abstract

The Middle American rust resistance gene *Ur-11* present in common bean (*Phaseolus vulgaris* L.) confers resistance to all but one known race of the pathogen *Uromyces appendiculatus* (Pers.) Unger. Even though progress has been made in understanding the host–pathogen interactions between common bean and *U. appendiculatus*, the causal alleles of the majority of rust resistance loci, including *Ur-11,* remain unknown. A genome-wide association study (GWAS) was conducted to identify genomic regions associated with resistance to the *U. appendiculatus* race 31–22, which is avirulent to *Ur-11* but virulent to other Middle American rust resistance genes. GWAS using genotypic data consisting of approximately 70,959 SNP markers and phenotypic data based on the median reaction type (1–9 scale) of a panel of 357 Middle American breeding lines and cultivars, plus 5 germplasm lines with the *Ur-11* locus derived from PI 181996, located *Ur-11* on chromosome Pv11. Twenty-seven SNP markers clustered in the 55.16–55.56 Mb region of the *P. vulgaris* UI111 reference. Multiple DNA sequence alignments detected a missense mutation [c.1,328A > G] in the *PvUI111.11G202400* gene model that encodes a leucine-rich repeat-containing protein in response to race 31–22. A PCR allele competitive extension marker (PACE) was developed and tested across a panel of ~ 700 Middle American dry bean genotypes. No recombination event was observed for the PACE marker among the tested genotypes; suggesting that the polymorphism on which it is based is very close to or in the *Ur-11* gene. This PACE marker will be a useful and reliable marker for marker-assisted selection of *Ur-11*-based resistance to bean rust.

**Supplementary Information:**

The online version contains supplementary material available at 10.1007/s00122-025-04836-9.

## Introduction

Rust, caused by *Uromyces appendiculatus* (Pers.:Pers.) Unger, is among the most damaging diseases of common bean (*Phaseolus vulgaris* L.) in humid tropical and subtropical production regions and is capable of causing periodic epidemics in temperate moist areas (Zaumeyer and Thomas 1957; Ballantyne 1974; Vargas 1980; Stavely and Pastor-Corrales 1989; Souza et al. 2013). The bean rust pathogen has a high virulence diversity with more than 90 races identified worldwide (Hurtado-Gonzales et al. 2017). Host plant resistance remains an effective and sustainable approach to managing *U. appendiculatus*, even in the face of its high variability (Osuna-Caballero et al. 2024).

It is believed that the *P. vulgaris*-*U. appendiculatus* pathosystem fits the gene-for-gene theory and involves dominant genes in the bean plant that confer monogenic resistance against various races of the rust pathogen (Stavely and Pastor-Corrales 1989; Montejo Domínguez et al. 2022). However, new findings suggest an oligogenic control of rust resistance in some common bean accessions (Leitão et al. 2023). At least 11 rust resistance loci have been identified and mapped to different linkage groups in the bean genome, and more resistance loci (named and unnamed) are yet to be mapped (Miklas et al. 2002, 2006; Steadman et al. 2002; Kelly et al. 2003; de Souza et al. 2011, 2013; Hurtado-Gonzales et al. 2017). Among mapped resistance loci, *Ur-3*, *Ur-3*^+^*, Ur-5*, *Ur-7*, *Ur-11,* and *Ur-14* belong to the Middle American gene pool, and *Ur-4, Ur-6, Ur-9, Ur-12*, and *Ur-13* are from the Andean gene pool (Steadman et al. 2002).

In 2008, a new *U. appendiculatus* race, 20–3, was identified in North Dakota, resulting in susceptibility of at least 27 of the most grown cultivars in North Dakota (Markell et al. 2009). Race 20–3 were virulent on the Middle American differential cultivars, Aurora (*Ur-3*) and GN1140 (*Ur-7*) and the Andean differential cultivars, Golden Gate Wax (*Ur-6*) and Montcalm (unknown gene). However, it is avirulant on the Middle American *Ur-11* gene. This indicates the importance of incorporating effective genes and pyramiding of those genes for long-term rust management in common bean.

The *Ur-11* gene provides broad-spectrum resistance against most *U. appendiculatus* races and is non-functional only against Honduran *U. appendiculatus* race 22–52 (formerly known as 108) (Pastor-Corrales et al. 2007; Wasonga et al. 2010; Hurtado-Gonzales et al. 2017). Therefore, the *Ur-11* gene in combination with other rust resistance genes can be used as the most cost-effective strategy for controlling the highly variable rust pathogen in common bean (Pastor-Corrales et al. 2007). The Guatemalan black beans PI 181996 and PI 190078 are known sources of the *Ur-11* gene in the Middle American gene pool (Pastor-Corrales et al. 2007). However, the *Ur-11* from PI 181996 was the one predominantly introgressed into common bean germplasm (Pastor-Corrales 2003). ‘ND-Falcon’ was the first pinto bean cultivar with the *Ur-11* derived from PI 181996 released by the NDSU dry bean breeding program in 2019 (Osorno et al. 2020).

Using BC_4_F_2_ individuals derived from backcrosses between NX-040*4 and PI 181996, the *Ur-11* locus was mapped to chromosome Pv11 and linked to two random amplified polymorphic DNA (RAPD) markers, OAC20_490_ co-segregating in coupling phase, and OAE19_890_ linked in repulsion phase at a distance of 6.2 ± 2.8 cM (Johnson et al. 1995). NX-040 is a sister line of 'Norstar' navy bean (Grafton et al. 1993). The RAPD marker OAE19_890_ was later located 1.0 cM from the *Ur-11* in a F_2_ population derived from Ruda´ × BelMiDak-RR-3 cross (Alzate-Marin et al. 2004). Ruda´ is a carioca-type cultivar (Aragão and Rech 1997), and BelMiDak-RR-3 is a navy bean germplasm line possessing the *Ur-11* gene from PI 181996 (Stavely et al. 1994; Pastor-Corrales 2003). Later, Queiroz et al. (2004) converted OAE19_890_ into a sequence characterized amplified region (SCAR) marker sAE19_890_. It is worth noting that Blast searches in Phytozome (http://phytozome.jgi.doe.gov) reveal the reverse primer of sAE19_890_ (5’-CAGTCCCTAAAGTAGTTTGTCCCTA-3’) is conserved across genetic backgrounds including Middle American (*P. vulgaris* UI111 reference genome assembly v1.1, Pv11: 55,335,635..55335659) and Andean (*P. vulgaris* G19833 genome assembly v2.1, Pv11: 51,387,148..51387172). Still, the forward primer of sAE19_890_ (5’-CAGTCCCTGACAACATAACACC-3’) is likely specific for the Carioca background.

Genome-wide association study (GWAS) is a powerful approach to detect genome–phenotype associations and validate loci identified by other methods. Recently, a GWAS on a Middle American diversity panel mapped the *Ur-11* of PI 181996 to a genomic interval from 50.5 Mb to 52.2 Mb on Pv11 of *P. vulgaris* G19833 (Monclova-Santana 2019). This region contains multiple disease resistance genes such as leucine-rich repeats containing (LRR) genes and corresponds to a genomic region from 54 to 56 Mb on Pv11 of *P. vulgaris* UI111 v1.1.

Even though great progress has been made in mapping and identifying candidate genes for rust resistance at the *Ur-11* locus, causal variants and their contributions to developing resistance to rust pathogen remain unknown. This study aimed to i) validate genomic regions associated with the *Ur-11* locus conferring resistance to *U. appendiculatus* in Middle American beans, ii) determine a variant or set of variants in candidate genes that might identify it as the *Ur-11* gene, and iii) develop a gene-based marker that can be utilized in marker-assisted selection in early stages of a breeding program for rust resistance.

## Materials and methods

### Plant material

A panel of 362 genotypes consisting of preliminary (PYT) and advanced yield trial (AYT) lines from the North Dakota State University dry bean program, germplasm lines, and cultivars known to possess the *Ur-11* locus derived from PI 181996 and cultivars from the Middle American gene pool was evaluated for this study. The panel included 126 pinto, 94 black, 50 pink and small red, 50 great northern, and 42 navy bean genotypes. The germplasm lines included the pinto bean BelDakMi-RR-5 and the great northern beans BelMiNeb-RR-1, BelMiNeb-RR-2, BelMiNeb-RMR-3, and BelMiNeb-RMR-4, all developed by the United States Department of Agriculture, Agricultural Research Station at Beltsville, Maryland (Pastor-Corrales 2003). The cultivars with the *Ur-11* locus from PI 181996 were great northern Beryl R (PVP# 200,600,224), pink bean Pink Floyd (PVP# 200,500,211), and pinto bean NE2-09–3 developed by Dr. Carlos Urrea (University of Nebraska, Scottsbluff).

### Inoculum preparation and plant inoculation

Rust inoculum preparation and plant inoculation followed a protocol by Jochua et al. (2008) with a small modification. Briefly, 25 mg of urediniospores of *U. appendiculatus* race 31–22 (previously known as race 67) was suspended in 300 ml of Tween 20 solution (40 µl of Tween 20 per 1000 ml of distillate water) and used to inoculate the unifoliate leaves of 8-day-old plants using a Paasche VL Series airbrush (www.paascheairbrush.com). Following drying, inoculated plants were incubated in humidity chambers overnight in darkness with misting for 20 s every 30 min at 21 ± 1 °C to increase humidity and aid infection. Plants were then transferred to a greenhouse (14 h days at 24 ± 2 °C; 10 h night at 21 ± 2 °C) for disease development. The experiment consisted of an alpha-lattice design with 3 replicates (rep) and 43 incomplete blocks [(plastic trays of 50 cells (10 × 5)] within each rep. In each incomplete block, the susceptible check ‘Othello’ was included. Each experimental unit consisted of five plants, placed in its pot. Plants were inoculated once in each replicate.

### Disease reaction evaluation

The rust reaction was scored 14 days post-inoculation using the 1–9 scale as described by Schoonhoven and Pastor-Corrales (1987). According to this rating system, plants with no visible rust pustules were considered immune and scored 1. Plants showing tiny pustules on less than 2% of their unifoliate area received a score of 3 and fell into the resistant category. Small and intermediate pustules covering approximately 5% of the unifoliate area resulted in a score of 5 for intermediate resistance. Plants were scored susceptible (7) and highly susceptible (9) for having large pustules covering approximately 10% and more than 25% of their unifoliate leaves, respectively. Proc univariate in SAS 9.4 (SAS Institute, Cary, NC, US) was used to calculate descriptive statistics, including the median of the rust reaction values.

### DNA extraction and sequencing library preparation

Young trifoliate leaves from 2-week-old plants were harvested for DNA isolation. Genomic DNA was isolated from approximately 50 mg of leaf tissue using Mag-Bind® Plant DNA Plus Kit (Omega Bio-Tek, Norcross, GA, US) following the product manual (https://omegabiotek.com/product/mag-bind-plant-dna-plus-96-kit). DNA was quantified using a nanodrop and diluted to 50 ng/μl. Sequencing libraries were prepared following a *Phaseolus*-specific protocol developed by Schröder et al. (2016). In brief, DNA from each genotype was double digested with two restriction enzymes, MseI and Taqα1, and uniquely barcoded. Each library was sequenced in paired-end runs (2 × 150 bp) at HudsonAlpha Institute for Biotechnology (Huntsville, AL, US) using Illumina HiSeq 2500 Sequencing System in rapid-run mode.

### Single-nucleotide polymorphism dataset

The raw sequencing reads were processed to trim low-quality reads with less than 80 bp in length and a default quality threshold score of 20 using SICKLE (Joshi and Fass 2011). The ‘BWA-MEM’ algorithm (Li 2013) was used to align the quality sequence reads against the UI111 v1.1 reference genome. The aligned reads were sorted and indexed with SAMtools (Danecek et al. 2021). Read group information for each genotype, including library, platform, and platform unit, were added using Picard tools (http://broadinstitute.github.io/picard). SNP calling was implemented using the MultisampleVariantsDetector module embedded in the NGSEPcore_4.2.0 software with the -maxAlnsPerStartPos 100 parameter (Tello et al. 2023). Multiallelic SNPs were discarded and SNPs with a minimum read depth ≥ 3, were selected. Additionally, markers with less than 30% missing sites and 5% heterozygotes sites were imputed using Beagle 5.4 (Browning et al. 2018). Finally, a genotypic dataset of 70,959 high-quality SNPs were retained after applying 5% minor allele frequency (MAF), which were used for GWAS.

### Genome-wide association study

GWAS analysis was performed using phenotype and genotype data with Genome-wide Efficient Mixed Model Analysis (GEMMA) (Zhou and Stephens 2012) software implementing a single-locus linear mixed model. Principal component analysis (PCA) was performed to estimate population structure. Population relatedness (kinship matrix) was generated using the GEMMA algorithm for centered relatedness. The linear mixed model was implemented including the first three PCA for population structure and kinship matrix for genetic relatedness. Manhattan and quantile–quantile (QQ) plots were generated using the R package qqman (Turner 2018). The marker-trait association was determined statistically significant based on the *p*-value (-log10(*p*)) detection threshold using the Bonferroni correction to control the genome-wide false-positive rate (*α* = 0.05). The proportion of phenotypic variance explained by the significant SNPs (R^2^) was calculated in TASSEL (Bradbury et al. 2007).

### Identification of candidate genes and sequence alignment

The flanking sequences from the significant SNP markers associated with the *Ur-11* locus were extracted with Integrative Genomics Viewer (https://igv.org/doc/desktop) and used in a BLAST search against the *P. vulgaris* UI111 v1.1 in Phytozome 13 (https://phytozome-next.jgi.doe.gov). LD blocks surrounding the significant SNPs identified by GWAS were defined according to the solid spine method of linkage disequilibrium (LD) and the extended spine if D’ > 0.8, implemented in the Haploview v4.1 software (Barrett et al. 2005). The defined LD block genomic regions were used for the candidate gene search. If a significant SNP was not located in the LD block, candidate genes were identified within 100 kb upstream and downstream of the peak SNPs' physical location (bp). Multiple DNA sequence alignments and variant reviews were performed with IGV. Translation of DNA sequence to protein sequence was carried out with Expasy (https://web.expasy.org/translate). Multiple protein sequence alignments were performed with Clustal Omega (https://www.ebi.ac.uk/jdispatcher/msa/clustalo).

### PACE genotyping assay

PCR Allele Competitive Extension (PACE) genotyping assays (allele-specific forward and common reverse primers) designed by Integrated DNA Technologies, Inc. investigated target SNP sites in exons and upstream regulatory regions of candidate genes. PACE SNP genotyping was performed with 20 ng of high-quality genomic DNA samples from the genotypes using the PACE 2.0 Genotyping Master Mix (Standard ROX—150 nM, 3CR Bioscience) in the presence of two competitive allele-specific forward primers and a common, reverse primer in a final volume of 8 μL (Table S1). The PCR amplification condition was 15 min at 94 °C for the hot start activation, 10 cycles of 20 s at 94 °C, 65 °C for 60 s (dropping 0.8 °C per cycle), then 38 cycles of 20 s at 94 °C and 60 s at 57 °C followed by a final point read of the fluorescence for 2 min at 22 °C on a CFX Opus 96 real-time PCR system and using the CFX Maestro Software (BIO-RAD).

## Results

Phenotypic reactions of breeding lines and cultivars to *U. appendiculatus* race 31–22 were consistent across replicates under greenhouse conditions. Median genotype scores ranged from highly resistant (1) to highly susceptible (9). In total, 24 (~ 7%) genotypes showed an immune reaction, 10 genotypes (~ 3%) were resistant, 25 genotypes (~ 7%) exhibited an intermediate reaction, and 292 genotypes (~ 81%) were susceptible or highly susceptible. No values were recorded for 11 (~ 3%) due to poor germination (Table S2). The germplasm lines and cultivars with the rust resistance *Ur-11* locus including great northern beans BelMiNeb-RR-1, BelMiNeb-RR-2, BelMiNeb-RMR-3, BelMiNeb-RMR-4, and Beryl R; pinto bean BelDakMi-RR-5 and NE2-09–3; and pink bean Pink Floyd were among the 24 genotypes that expressed an immune reaction to race 31–22. Fourteen slow-darkening pinto, one black, one navy, and one pink bean breeding lines also showed an immune response. Seven black, one navy, and one slow-darkening pinto bean breeding lines were resistant.

**Fig. 1:**
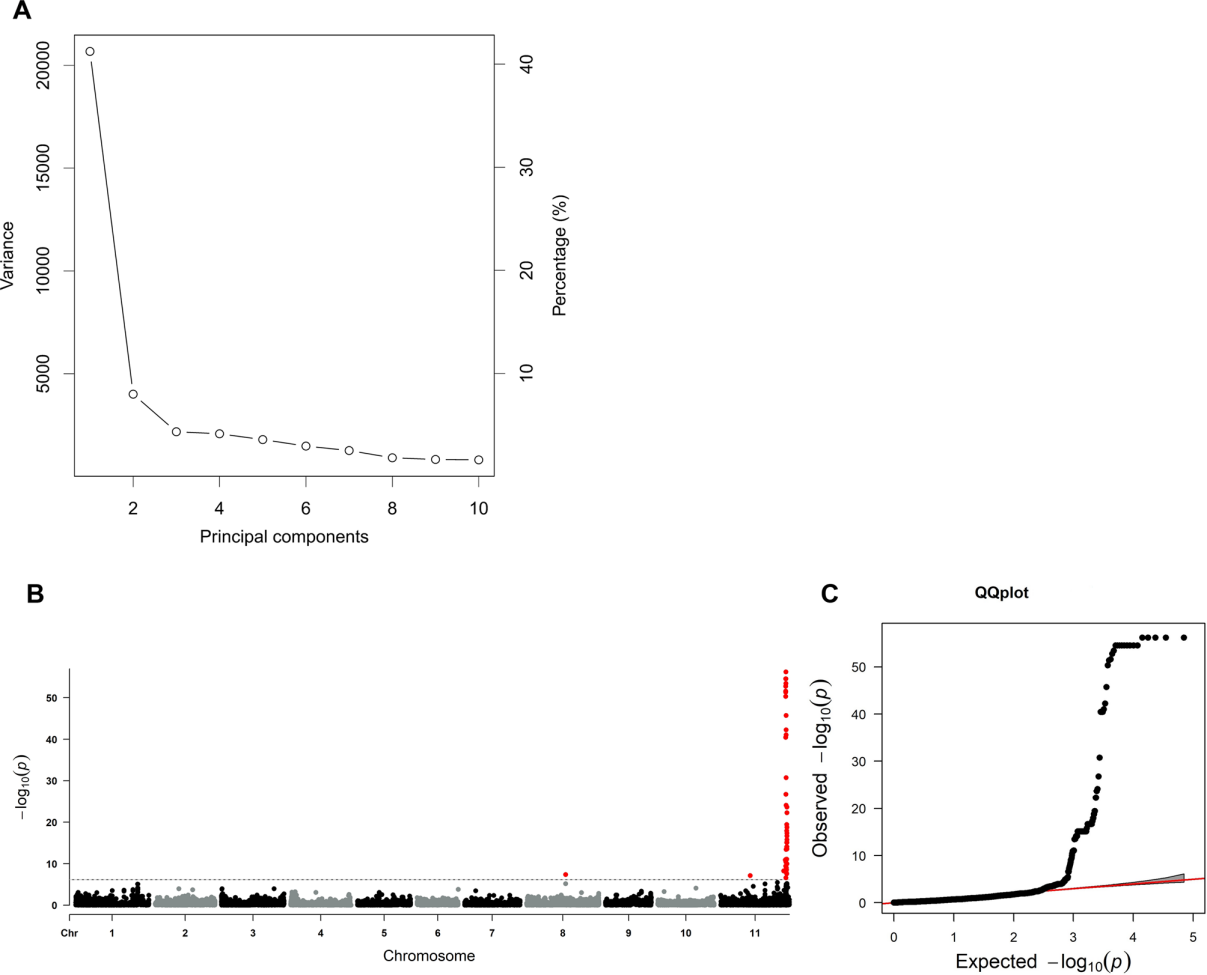
**A** Manhattan plot highlighting SNPs associated with the *Ur-11* locus in the 55.16–56.33 Mb region of Pv11 of the *P. vulgaris* UI111 reference genome assembly v1.1, with **B** QQ plot showing substantial deviation from the diagonal for highly trait-associated SNPs. *P. vulgaris* chromosomes (1–11) are represented on the x-axis, and a -log 10 (p) values are shown on the y-axis. The red line indicates the threshold at a significance value of -log10(*p*) = 4.7

GWAS was conducted using genotypic data consisting of 70,959 SNP markers (Table S2) and phenotypic data based on the median reaction type (1–9 scale) of 362 middle American-type bean genotypes (Table S3). Population structure was constructed by PCA which revealed that the first and second PCA acconted for 41.23 and 7.99% variance, respectively. The first three PCs explained about 53.56% of variance and the inflection point occurred at the first three PCA (Fig. 1A); therefore, we included three PCA in the GWAS model to control population structure. Based on the Bonferroni correction at 5% experiment-wise error rate, the significant threshold was *P* ≤ 7.05E-07; LOD ≥ 6.2 (Fig. 1B). GWAS revealed 86 significant SNP markers, one SNP on chromosome Pv08 and the remainder on Pv11 of the *P. vulgaris* UI111 reference genome assembly v1.1 (Fig. 1A, B; Table S4). Nineteen LD blocks were defined around the 86 significant SNPs associated with *Ur-11* (Table S4). From these, 30 SNP markers within a distinct peak on chromosome Pv11 in the 55.16–56.21 Mb region explained a higher proportion of phenotypic variance for resistance to race 31–22 than the others. Within the SNP peak, S11_55167465 and S11_55403490 showed the strongest association with the trait explaining 41.73% of the phenotypic variance (Table 1). The peak SNPs in this cluster are bordered upstream and downstream by gene models that encode proteins in response to abiotic and biotic stimuli, including NBS-LRR proteins (Table S6). Another SNP marker, S11_25155352, located at position 25,155,322 bp on chromosome Pv11 explained 8.1% of the phenotypic variance. It was approximately 100 bp upstream of gene model *PvUI111.11G124200* that encodes an ATP-dependent clp protease proteolytic subunit 1. On chromosome Pv08, S08_33204501 explained 8.1% of the phenotypic variance and was located approximately 70 kb upstream of gene model *PvUI111.08G148500* that encodes a ribosomal protein S6 kinase (S6K). Of the 30 peak SNPs, four SNP markers (S11_55167465, S11_55167606, S11_55167642, and S11_55167692) were found at approximately 14.2 to 22.8 kb upstream of the gene models *PvUI111.11G202300* and *PvUI111.11G202400* that encode NBS-LRR proteins. Five SNP markers (S11_55220618, S11_55220638, S11_55220675, S11_55221024, and S11_55221035) were found near the NBS-LRR protein encoding genes *PvUI111.11G202600*, *PvUI111.11G202700, PvUI111.11G202800, PvUI111.11G203000, PvUI111.11G203100,* and *PvUI111.11G203200* and a receptor protein kinase encoding gene *PvUI111.11G203300*. The gene models *PvUI111.11G203600, PvUI111.11G205100, PvUI111.11G205700, PvUI111.11G206000, and PvUI111.11G206100* were other NBS-LRR genes around the peak SNPs within the genomic region (Tables 1 and S6).

**Table 1:** Significant single-nucleotide polymorphism (SNP) markers on chromosomes Pv08 and Pv11 from a genome-wide association study (GWAS) of preliminary and advanced breeding lines and cultivars from the North Dakota State University dry bean breeding program, plus germplasm lines from the United States Department of Agriculture, Agricultural Research Station at Beltsville, Maryland, in association with *Uromyces appendiculatus* races 31–22. SNP marker positions are relative to the *P. vulgaris* UI111 reference genome assembly v1.1

Reference SNP	Chromosome	Position (bp)	−log_10_(*p*)	Variance explained (%)
S08_33204501	8	33,204,501	7.4	8.1
S11_25155352	11	25,155,352	7.1	8.1
S11_55397512	11	55,397,512	56.2	41.73
S11_55397514	11	55,397,514	56.2	41.73
S11_55397572	11	55,397,572	56.2	41.73
S11_55397575	11	55,397,575	56.2	41.73
S11_55466915	11	55,466,915	56.2	41.73
S11_55167465	11	55,167,465	54.5	41.73
S11_55167606	11	55,167,606	54.5	41.73
S11_55401744	11	55,401,744	54.5	41.73
S11_55401745	11	55,401,745	54.5	41.73
S11_55401746	11	55,401,746	54.5	41.73
S11_55401884	11	55,401,884	54.5	41.73
S11_55401904	11	55,401,904	54.5	41.73
S11_55403439	11	55,403,439	54.5	41.73
S11_55403490	11	55,403,490	54.5	41.73
S11_55458849	11	55,458,849	53.4	41.00
S11_55167642	11	55,167,642	52.8	40.00
S11_55220618	11	55,220,618	51.6	39.00
S11_55167692	11	55,167,692	51.4	38.00
S11_55220638	11	55,220,638	50.3	38.05
S11_55564761	11	55,564,761	45.7	37.70
S11_55565283	11	55,565,283	42.2	36.00
S11_55564702	11	55,564,702	41.0	33.81
S11_55220675	11	55,220,675	40.5	31.88
S11_55221024	11	55,221,024	40.5	31.88
S11_55221035	11	55,221,035	40.5	31.88
S11_55565595	11	55,565,595	30.7	30.66
S11_55374026	11	55,374,026	26.7	28.18
S11_55565511	11	55,565,511	24.1	26.53
S11_56214001	11	56,214,001	23.6	24.83
S11_56214007	11	56,214,007	22.3	24.48

**Fig. 2:**
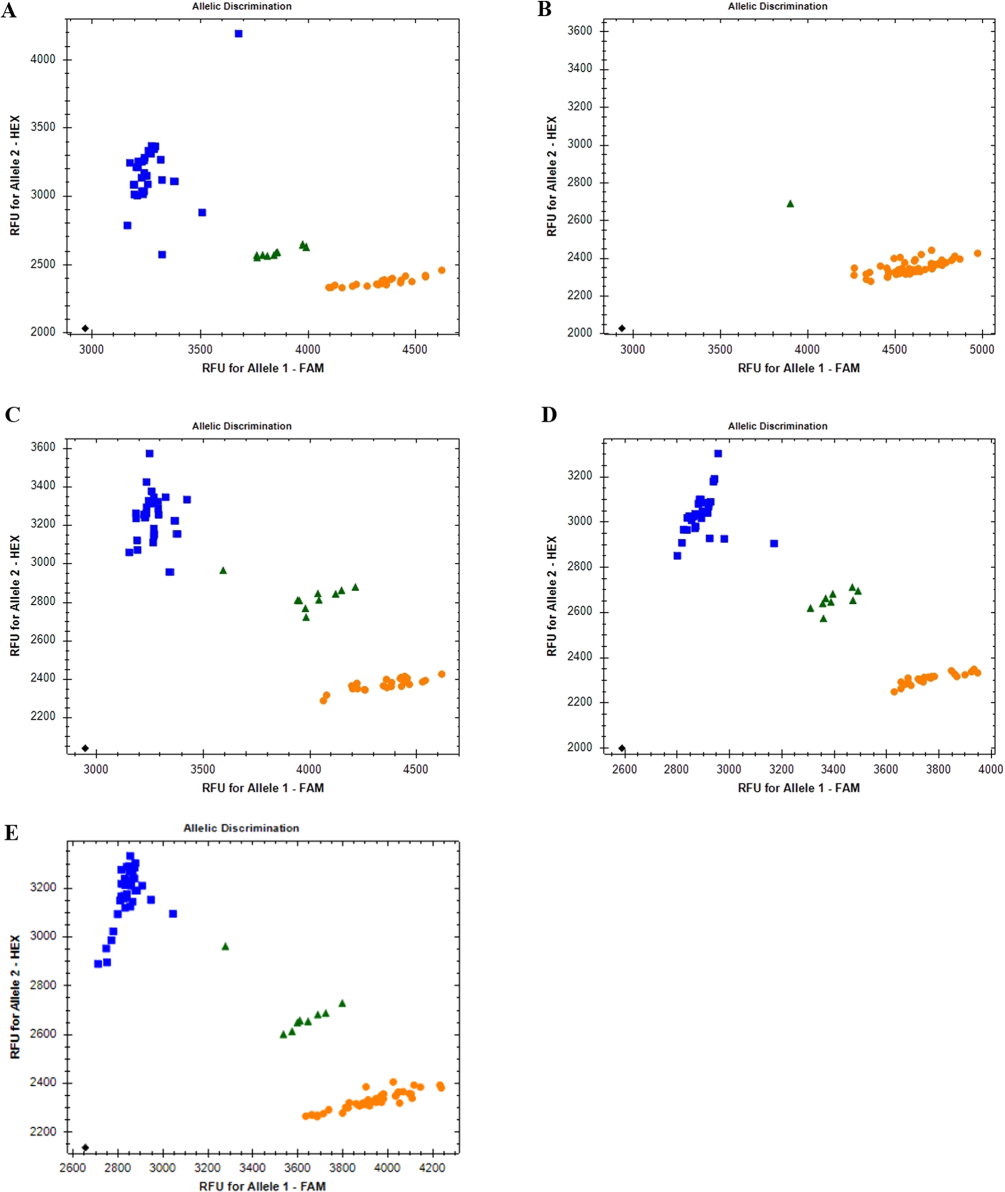
Bio-Rad CFX Maestro images of four different PCR Allele Competitive Extension (PACE) genotyping assays linked to the rust resistance *Ur-11* locus and comparison of their efficiency to differentiate the respective phenotypes in 63 navy bean genotypes with an immune reaction to rust race 31–22. Orange circles have a FAM-type allele and represent resistant genotypes homozygous for allele 1; blue squares have a HEX-type allele and represent susceptible genotypes homozygous for the reference allele 2; green triangles represent heterozygotes; and black diamonds represent no-template controls. FAM and HEX signals are reported in relative fluorescence units (RFUs). Fluorescence intensities were normalized using a passive reference dye (ROX). **A** PACE marker S11_55182817 represents a single-nucleotide substitution in the exon [c.1,155C/T] of *PvUI111.11G202300*. **B** PACE marker S11_55191718 represents a single-nucleotide substitution in the exon [c.1,328A > G] of *PvUI111.11G202400*. **C** PACE marker S11_55458849 represents a single-nucleotide substitution in the exon [c.610G > A] of *PvUI111.11G204900*. **D** PACE marker S11_55482888 represents a single-nucleotide substitution in the exon [c.3,565G > A] of *PvUI111.11G205100*. **E** PACE marker S11_55997953 represents a single-nucleotide substitution in the exon [c.148G > A] of *PvUI111.11G209200* in UI111 reference genome and corresponds to S11_51904022 in *Phvul.011G203300* of G19833 reference genome

DNA sequence alignments of the candidate genes associated with the *Ur-11* in 13 genotypes that possess the *Ur-11* gene from PI 181996 and expressed an immune reaction to race 31–22, including BelMiNeb-RR-1, BelMiNeb-RR-2, BelMiNeb-RMR-3, BelMiNeb-RMR-4, BelMiNeb-RMR-5, BelMiDak-RR-1, BelMiDak-RR-2, BelDakMi-RR-5, Beryl R, Pink Floyd, NE2-09–3, PI 181996, and Topaz R (personal communication with Dr. Phil Miklas) with 26 susceptible genotypes, including pinto beans AC Island, Buster, CDC Camino, Chase, Croissant, Frontier, Lariat, Kimberly, La Paz, Maverick, Monterrey, Montrose, Nodak, SDIP-1, Sedona, Stampede, UI-114, USPT-WM-1, Windbreaker, great northern bean BelNeb-RR-1, small red beans AC Redbod, AC Scarlet, Merlot, Rosetta, and black beans Mexico 235 and Mexico 309 revealed single- and multiple nucleotide polymorphisms distributed within exons of these genes. In total, 12 PACE genotyping assays were designed for gene variants linked to the *Ur-11* locus (Table 2). The efficiency of the present study’s PACE markers and the PACE marker S11_51904022 currently in use (McClean, Miklas, and Pastor-Corrales, unpublished), in differentiating resistant genotypes with the *Ur-11* from PI 181996 from the ones with the *Ur-11* from PI 190078 and the susceptible genotypes was evaluated through their cross-validation in a set of ~ 700 bean genotypes composed of cultivars and breeding lines and ~ 300 Middle American Diversity Panel (Moghaddam et al. 2016) from different genetic backgrounds and populations. The PACE marker S11_51904022 represents a single-nucleotide substitution [c.148G > A] in the exon of gene model *Phvul.011G203300* that encodes a NADH dehydrogenase (ubiquinone) 1 alpha subcomplex subunit 6 in the *P. vulgaris* G19833 reference genome assembly v2.1. The S11_51904022 corresponds to S11_55997953 in the gene model *PvUI111.11G209200* of the UI111 reference genome (http://phytozome.jgi.doe.gov). The results showed no consistent association between the gene variants and plant reaction type for most of the detected polymorphisms in the candidate genes across the genotypes. However, the PACE marker S11_55191718 which represents a single-nucleotide substitution in the exon [c.1,328A > G] of the candidate gene *PvUI111.11G202400* (Table 2) did precisely differentiate the resistant genotypes with the *Ur-11* from two different sources and susceptible genotypes across different populations including two biparental navy bean populations derived from crosses between Puerto Rican bean lines 2104–1-1 × PR0806-81 and 2104–1-2 × PR0806-81 (made by Dr. James Beaver at the University of Puerto Rico and advanced to homozygosity at NDSU). Table S5 compares the efficiency of PACE markers S11_55191718 and S11_55997953 in distinguishing the dry bean genotypes with the *Ur-11* from PI 181996 and the susceptible genotypes. The parental lines 2104–1-1 and 2104–1-2 are navy bean genotypes with the rust resistance *Ur-5* gene (personal communication with Dr. Beaver). The parental line PR0806-81 (Reg. No. GP-297, PI 672995) is a navy bean germplasm line possessing the *Ur-11* gene from PI 181996 (Beaver et al. 2015). Figure 2 demonstrates the outputs of four PACE assays for 63 navy bean breeding lines with an immune reaction to rust race 31–22. The PACE marker S11_55191718 cosegregated with the *Ur-11* resistance phenotype compared to markers S11_55182817, S11_55458849, S11_55482888, and S11_55997953.

**Table 2:** List of PCR Competitive Extension (PACE) genotyping assays designed for genetic variants in the exons of candidate genes linked to the *Ur-11* locus on chromosome Pv11 of the UI111 reference genome that consistently differentiates genotypes with immune reactions to rust race 31–22 from the susceptible genotypes based on the results of DNA sequence alignments

Gene	SNP physical position (bp) UI111 genome	Reference allele	Alternate allele	Marker ID	Primer type	Primer sequence
PvUI111.11G202300	55,182,814	C	G	S11_55182814	Alternate^1^	FAM-GCCTTGAAAACAATGGGAAGTCTAC
					Reference^2^	HEX-GCCTTGAAAACAATGGGAAGTCTAG
					CR^3^	TGCTTTCCCATTCCCAAAAGGATGATTTA
PvUI111.11G202300	55,182,817	C	A	S11_55182817	Alternate	FAM-CCATTCCCAAAAGGATGATTTACTGTT
					Reference	HEX-CCATTCCCAAAAGGATGATTTACTGTG
					CR	TAGCCTTGAAAACAATGGGAAGTCTASTA
PvUI111.11G202300	55,182,818	A	G	S11_55182818	Alternate	FAM-CCATTCCCAAAAGGATGATTTACTGC
					Reference	HEX-CCATTCCCAAAAGGATGATTTACTGT
					CR	CTTTAGCCTTGAAAACAATGGGAAGTCTAGTA
PvUI111.11G202400	55,191,672	G	T	S11_55191672	Alternate	FAM-CCTTCCTTCTTATCTGAAGAAATGCTTTT
					Reference	HEX-CCTTCCTTCTTATCTGAAGAAATGCTTTG
					CR	CACATAACCTTTGGGAAATAAGGCACAAA
PvUI111.11G202400	55,191,718	A	G	S11_55191718	Alternate	FAM- AAAGGTTATGTGTTTGACAAGGAGTG
					Reference	HEX- AAAGGTTATGTGTTTGACAAGGAGTA
					CR	CACATAACCTTTGGGAAATAAGGCACAAA
PvUI111.11G202400	55,192,034	G	T	S11_55192034	Alternate	FAM-CAGTCAGTTGGCATAAATGTATGCAAA
					Reference	HEX- CAGTCAGTTGGCATAAATGTATGCAAC
					CR	GGGTTTGGAAGTTTGGTTGATACTCAAAA
PvUI111.11G202400	55,192,298	C	T	S11_55192298	Alternate	FAM-AAGCTCCTTCAATCTTCGACAATCA
					Reference	HEX-AAGCTCCTTCAATCTTCGACAATCG
					CR	AAACTACCTGACTCCATAAGTTTACTCAAA
PvUI111.11G204900	55,458,849	G	A	S11_55458849	Alternate	FAM-CAGTGTCTGTGGCTGTTGGTA
					Reference	HEX-CAGTGTCTGTGGCTGTTGGTG
					CR	GGATCTGCCATCTCATTGGGAAGAA

Gene	SNP physical position (bp) UI111 genome	Reference allele	Alternate allele	Marker ID	Primer type	Primer sequence
PvUI111.11G204900	55,459,336	G	A	S11_55459336	Alternate	FAM-GGTATTTGCCGGTTGAAGTGAA
					Reference	HEX- GGTATTTGCCGGTTGAAGTGAG
					CR	CAACCAAAACACTTCGCATGCCCAT
PvUI111.11G205100	55,482,888	G	A	S11_55482888	Alternate	FAM-CTCCAACGCCTACCAGAGGAGA
					Reference	HEX-CTCCAACGCCTACCAGAGGAGG
					CR	GCAAAGGACACTGCTTTATTGTAAGACTT
PvUI111.11G205100	55,483,738	C	G	S11_55483738	Alternate	FAM-GTCCAACTGCAGCTTTCCACAC
					Reference	HEX-GTCCAACTGCAGCTTTCCACAG
					CR	CACTCTCAGAGCTCTTGATTTAGAACTA
PvUI111.11G205100	55,484,893	C	A	S11_55484893	Alternate	FAM-GAGTTGATGAACCAAAAGGTATACCA
					Reference	HEX-GAGTTGATGAACCAAAAGGTATACCC
					CR	GGTTGTAGATGACAAATGACGGATTGTTT

^1^Resistant allele

^2^Susceptible allele

^3^Common reverse

**Fig. 3:**
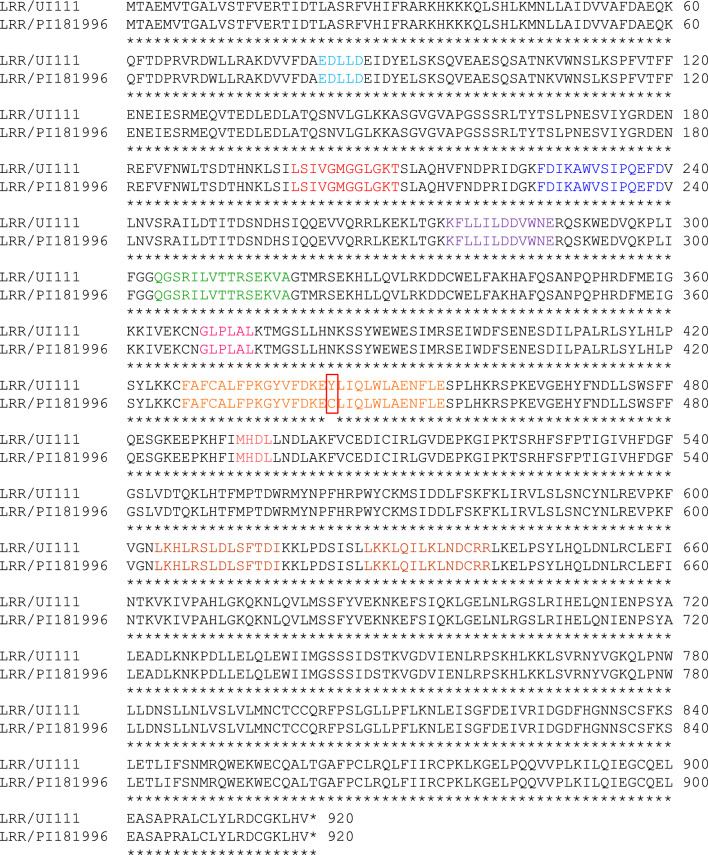
Sequence alignment of NBS-LRR protein encoded by *PvUI111.11G202400* in *P. vulgaris* UI111 reference genome (rust-susceptible genotype) and the putative translation of the homolog of *PvUI111.11G202400* in PI 181996 (possessing the rust resistance *Ur-11* gene). A substitution of cysteine (C) for tyrosine (Y) at position 443 (bordered by red lines) was detected in the protein sequence of the *PvUI111.11G202400* in PI 181996 (rust-susceptible genotype). Characteristic motifs of CC-NBS-LRR are identified, including EDLLD motif (teal residues) in the CC domain, P-loop (red residues), RNBS-A (blue residues), kinase-2 (purple residues), RNBS-B (green residues), GLPLAL (megneta residues), RNBS-D (orange residues), and MHDL (pink residues) in the NBS domain, plus the LKKLQILKLNDCRR motifs (brown residues) in the NBS-LRR domain. A substitution of cysteine (C) for tyrosine (Y) at position 443 (bordered by red lines) was detected in the protein sequence of the *PvUI111.11G202400* in PI 181996

A blastp analysis found *PvUI111.11G202400* exhibited about 70% sequence identity with coiled-coil nucleotide-binding site-leucine-rich repeat (CC-NBS-LRR) type disease resistance proteins such as At3g14460, RGAs, RPG1-B, and RPP13-like proteins in legume crops *Vigna angularis* (Wild.) (adzuki bean), *V. radiata* var. radiata (mung bean), *V. umbellata* (Thunb.) Ohwi & H.Ohashi (ricebean), *V. unguiculata* (L.) Walp. (cowpea), *Glycine max* (L.) Merr., and *G. soja* Siebold & Zucc. across its entire query length. Multiple sequence alignment of CC-NBS-LRR proteins and putative NBS-LRR proteins encoded by the gene homologs of *PvUI111.11G202400* in UI111 and PI 181996 (McClean et al. 2022) revealed characteristic motifs of a typical CC-NBS-LRR for the *PvUI111.11G202400* protein. Figures 3 and S1 show that the *PvUI111.11G202400* protein comprises motifs with identical or similar residues to its putative homologs in different species. In order from the N-terminus, the protein has a conserved EDLLD motif in the CC domain; P-loop (also called kinase 1 or Walker A site, GGVGKT), RNBS-A (KAWVCVSD), kinase-2 (also called Walker B site, LVLDDV), RNBS-B (also called kinase-3a, NGCKVLFTTRSEEVC), GLPL (GLPLAL), RNBS-D (CFLYCALF), and MHDV (MHDV) in the NBS domain (also called the NB or NB-ARC); and LKKLQILKLNDCRR motifs in the LRR domain (Hammond-Kosack and Jones 1997; Meyers et al. 1999, 2003; López et al. 2003; McHale et al. 2006; Rairdan and Moffett 2006; Rairdan et al. 2008; Wang et al. 2015; Wu et al. 2017; Liu et al. 2019; Goyal et al. 2020).

The *PvUI111.11G202400* c.1,328A > G polymorphism results in a substitution of tyrosine (Y) with a cysteine (C) at position 443 (Y443C) for the rust-resistant genotype PI 181996 (Fig. 3). Therefore, the protein encoded by *PvUI111.11G202400* in the rust-susceptible genotype UI111 would be expected to have identical CC and LRR domains to its counterpart in PI 181996. Still, the RNBS-D motif in the NBS domain shows enrichment for cysteine in the resistant *PvUI111.11G202400* protein and a slight increase in the protein sequence identity with its homologs in other species due to the amino acid substitution (Fig. S1). PACE marker S11_55191718 was developed to differentiate the resistance allele from the susceptible one in the *PvUI111.11G202400*. The primer sequences were designed to bind a site that ends with the G at c.1,328 that is A in the susceptible genotypes (Table 2). The marker was tested across a panel of ~ 700 Middle American dry bean genotypes. No recombination event was observed for the marker among this population indicating that the polymorphism on which it is based is very close to or in the *Ur-11* gene.

## Discussion

Introgression of *Ur-11,* the most effective gene against the highly variable common bean rust pathogen, into dry beans has gained increased attention recently. The lack of reliable molecular markers has hindered the effective selection of lines possessing the *Ur-11* gene in bean breeding programs. Here it is shown that *Ur-11* is physically located close to gene model *PvUI111.11G202400* which encodes an NBS-LRR protein located on chromosome Pv11. This model is near the reverse primer of SCAR marker sAE19890 which was previously found to be linked to the *Ur-11* locus (Queiroz et al. 2004) and in the same genomic interval associated with an immune response to three *U. appendiculatus* races that attack *Ur-11* alleles (Monclova-Santana 2019). In agreement with Monclova-Santana (2019), we found that most candidate genes tagged by the *Ur-11* haplotype response to race 31–22 encode NBS-LRR proteins (Table S6) that constitute the largest protein family encoded by plant disease resistance (R) genes in response to bacterial, fungal, and viral pathogens (Wu et al. 2017).

A common NBS-LRR consists of a diverse N-terminal domain, a central NBS domain, and a C-terminal LRR domain. Plant NBS-LRR proteins can be divided into two subfamilies based on the presence of Toll/interleukin-1 receptor (TIR) or CC (or non-TIR) domains in the N-terminal domain (Bentham et al. 2018). The N-terminal TIR and CC, NBS, and LRR domains have different roles during host–pathogen recognition. Both TIR and CC domains are thought to be the receptor modules required for downstream signal transduction post-NBS-LRR activation (Takken and Goverse 2012); however, CC domains from a variety of different NBS-LRRs have also been implicated in guardee or effector perception (Khan et al. 2016). The highly conserved NBS domain typically consisting of ~ 300 amino acids is a functional ATPase domain, and its nucleotide-binding state is proposed to regulate the activity of the R protein (Ooijen et al. 2008).

Six conserved motifs have been identified in the NBS domain of CC-NBS-LRR proteins, including P-loop, kinase-2, RNBS-B, GLPL, RNBS-A, and RNBS-D (He et al. 2022). The functions of these conserved motifs are not well known, but it is believed that they may play roles in binding ATP for the regulation of protein activity (Takken et al. 2006). Mutations in the CC-NBS-LRR gene *Pm21* which confers effective resistance to wheat (*Triticum aestivum* L.) powdery mildew (caused by *Blumeria graminis* f. sp. *Tritici*) (He et al. 2018) resulted in amino acid substitutions in or near the RNSB-D (L414F, P415L, L418F, R419H, P420S, and C421Y), leading to loss-of-function (He et al. 2022). In *Arabidopsis thaliana*, *RMP1* encodes a CC-NBS-LRR protein in cell plasma membranes in response to the phytopathogenic bacterium *Pseudomonas syringae* (Boyes et al. 1998). Two amino acid substitutions in or next to the RNSB-D motif (S439F and P442L) of RPM1 impaired the protein function (Tornero et al. 2002). Substitutions of two amino acids (L456P/Y458H) in or next to the RNBS-D resulted in an extended resistance spectrum to wheat powdery mildew conditioned by of the CC-NBS-LRR gene *Pm3f* (Stirnweis et al. 2014). Amino acid changes in or near RNBS-D motifs were suggested to impair the ATP/ADP binding state of the CC-NBS-LRR protein encoded by *Zea mays Rp1-D21*, which confers resistance against *Puccinia sorghi*, the causal agent of maize common rust (Wang et al. 2015). In potatoes (*Solanum tuberosum* L.), similar observations were reported for the NBS-LRR proteins encoded by *Rx1*, which confers resistance to potato virus X, and the *Gpa2* gene that confers resistance to the potato cyst nematode *Globodera pallida* (Bendahmane et al. 2002; Slootweg et al. 2013).

Our results suggest that a missense mutation [c.1,328G > A] in the UI111 allele of *PvUI111.11G202400* likely causes an amino acid substitution (C443Y) in or next to the RNBS-D motif that disrupts the protein function in a similar way to the effect of amino acid substitution (C421Y) in the wheat CC-NBS-LRR gene *Pm21*. This similarity may indicate the importance of the cysteine (C) residues in CC-NBS-LRRs (He et al. 2022). We speculate that the wild-type allele of the *PvUI111.11G202400* gene is associated with rust resistance conditioned by the *Ur-11* locus.

In the present study, the use of rust pathogen race 31–22 which is avirulent to the *Ur-11* locus but is virulent to many other Middle American *Ur* loci including the *Ur*-3 locus, which is closely linked to *Ur-11* (Hurtado-Gonzales et al. 2017), plus the phenotypic data obtained from the responses of the Middle American genotypes to race 31–22 suggests more accurate and reliable results than the previous studies (Monclova-Santana 2019). The gene-based PACE marker S11_55182817 was more efficient in distinguishing the resistant genotypes with the *Ur-11* from PI 181996 and susceptible genotypes within different Middle American market classes and populations as compared to the PACE marker S11_55997953 currently in use. This new marker would enable bean breeders to select for bean genotypes carrying the dominant allele from those possessing recessive alleles at the *Ur-11* locus in early generations of population development, thus preventing the need for plant inoculation.

In addition to peak SNPs on chromosome Pv11, we found SNP S08_33204501 on Pv08 and SNP S11_25155352 on Pv11 significantly associated with reaction to race 31–22 which have not been associated with any known rust resistance genes. SNP S08_33204501 was 70 kb upstream of the *PvUI111.08G148500* gene that encodes a ribosomal S6K that regulates cell growth, cell proliferation, and stress response via modulating protein synthesis and ribosomal biogenesis (Obomighie et al. 2021). SNP S11_25155352 was approximately 100 bp upstream of gene model *PvUI111.11G124200* that encodes an ATP-dependent clp protease proteolytic subunit 1 that plays a significant role in protein quality control and homeostasis, particularly in the chloroplast (Sjögren and Clarke 2011).

Future work to validate the role of CC-NBS-LRR gene *PvUI111.11G202400* in rust resistance might include targeted mutation of the amino acid 443 in the NBS domain of the protein, followed by *Agrobacterium*-mediated transient expression in bean cotyledons following procedures described by Williams et al. (2011) for flax rust resistance genes.

## Supplementary Information

Below is the link to the electronic supplementary material.

Supplementary file1 (XLSX 92621 kb)
